# Measuring cytokines in quarter milk samples with a fluorescent bead-based multiplex assay

**DOI:** 10.3168/jdsc.2026-1038

**Published:** 2026-06-11

**Authors:** Anja Sipka, Matthias Wieland

**Affiliations:** Department for Population Medicine and Diagnostic Sciences, Cornell University, Ithaca, NY 14853

## Abstract

Mastitis is a highly prevalent, costly disease affecting well-being and productivity in dairy cows. Management of intramammary infections typically relies on aerobic culture for identification of a causative pathogen. Cytokines regulate inflammatory processes in the infected quarter. Their production and secretion patterns are influenced by the invading pathogen and by the host's immune system. A previously validated fluorescent bead-based multiplex assay was used for the quantification of bovine IL-10, TNF-α, and IFN-γ. Our goal was to demonstrate that this assay can be used for milk samples and to measure concentrations of the 3 analytes in quarter milk samples with different bacteriological culture results. We enrolled quarter milk samples that were submitted for routine mastitis diagnostics by farm personnel from commercial dairies (n = 17) in central New York. We selected samples identified with major mastitis pathogens, including coliform bacteria (n = 51; termed the coliform group, comprising *Escherichia coli* and *Klebsiella* spp.), bacteria from the *Streptococcus* family (n = 53; Strep group: *Streptococcus uberis, Streptococcus dysgalactiae*, and *Lactococcus* spp.), *Staphylococcus aureus* (n = 36; *S. aureus* group), samples from mastitic quarters without bacterial growth after 48-h aerobic culture (n = 44; no growth), and samples from healthy quarters (n = 55; healthy). When comparing cytokine concentrations for different bacteriological culture results, we found that the coliform group had the highest IL-10 and TNF-α concentrations compared with all other bacteriological culture results. The Strep group had higher IFN-γ and TNF-α concentrations compared with *S. aureus*, no growth, and healthy samples, and higher IL-10 concentrations compared with no growth and healthy quarters. No significant differences were found between *S. aureus*, no growth, and healthy samples, or between no growth and healthy samples. In this study we observed differences in cytokine concentration patterns across the selected bacteriological culture results. We demonstrated that the bovine multiplex assay can be used to measure IL-10, TNF-α, and IFN-γ in milk samples and could provide a useful tool for screening milk samples and furthering our understanding of inflammation dynamics in mastitis. Although differences were found between cytokine concentrations and bacteriological culture results, they showed high individual variation, and future studies should include additional information on enrolled samples such as occurrence of clinical signs, treatment, and resolution of infection.

## Highlights

• A multiplex assay was used to measure TNF-α, IL-10, and IFN-γ in quarter milk samples.
• Quarter milk samples with different culture outcomes were enrolled.
• Differences were found between culture outcomes with high individual variation.
• The multiplex assay is an efficient tool to screen milk samples for cytokines.

## Graphical Abstract

**Figure:**
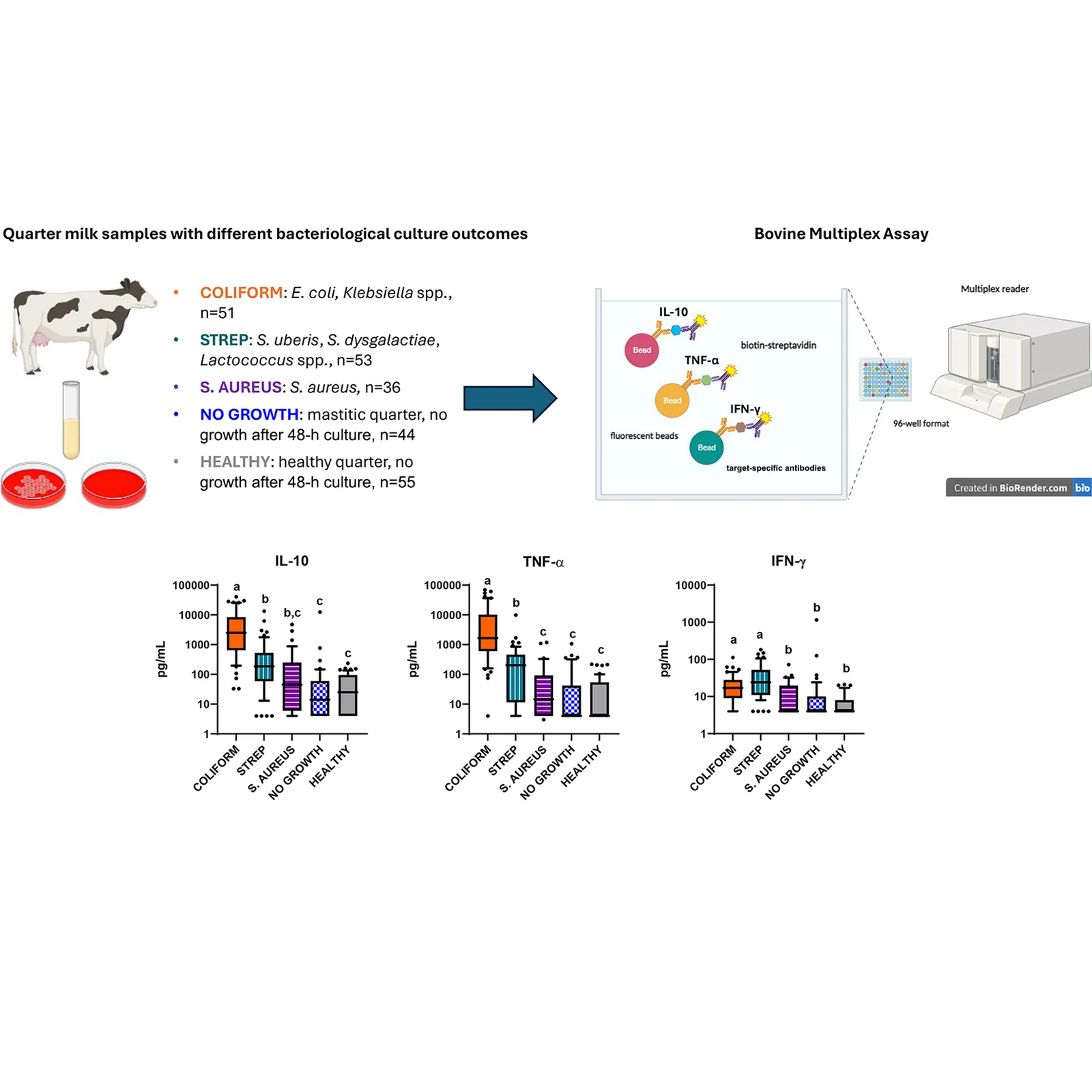
**Summary:** Management of intramammary infections typically relies on aerobic culture for the identification of a causative pathogen that is associated with some limitations (time, sample quality, lack of bacterial growth). Our goal was to measure concentrations of cytokines (IL-10, TNF-α, and IFN-γ) in quarter milk samples with different culture outcomes using a fluorescent bead-based multiplex assay (bovine multiplex assay). We enrolled quarter milk samples identified with major mastitis pathogens (coliform bacteria, *Streptococcus* family, *Staphylococcus aureus*) as well as mastitic samples with no growth after 48 hours of aerobic culture and samples from healthy quarters (somatic cell count <200,000 cells/mL). We found differences between culture outcomes, but also a high individual variation between samples. The bovine multiplex assay can potentially be a useful tool for the quantification of IL-10, TNF-α, and IFN-γ in quarter milk samples, facilitating high-throughput screening for potential biomarkers for bovine mastitis.

Mastitis is one of the most prevalent diseases in dairy cows, negatively affecting the animals' well-being and leading to considerable economic losses for dairy farmers (Klaas and Zadoks, 2018). Current management recommendations for bovine mastitis are based on identification of the causative pathogen to ensure most prudent use of antimicrobials (de Jong et al., 2023a,b). Commonly identified mastitis pathogen groups include coliform bacteria (*Escherichia coli, Klebsiella* spp.), bacteria of the *Streptococcus* family (*Streptococcus dysgalactiae, Streptococcus uberis, Streptococcus agalactiae, Lactococcus* spp.) and *Staphylococcus aureus* (Vasquez et al., 2017; McDougall et al., 2018; Schmenger and Kromker, 2020). Aerobic culture is a widely used method for mastitis diagnosis and takes at least 24 h to obtain actionable results. In ∼20% of samples from quarters with clinical mastitis, no bacterial growth in aerobic culture is found (Fuenzalida and Ruegg, 2019). This could be due to the elimination of the invading pathogen by the host's immune system, the pathogen not being shed in milk in a sufficient concentration at the time of sampling, or the presence of a pathogen that does not grow under aerobic conditions.

During an intramammary infection the host immune system produces soluble immune mediators such as cytokines and chemokines. These play an instrumental role in trafficking leukocytes to the affected quarter, activating immune cells in the mammary gland, and restoring homeostasis (Bannerman, 2009; Aitken et al., 2011). Previous studies have investigated whether cytokine patterns can be associated with certain mastitis pathogens. Most of these studies were performed in experimental mastitis models, and only a few studies have included field samples from naturally infected quarters (Hagiwara et al., 2001; Hisaeda et al., 2001, 2011; Ohtsuka et al., 2001). The studies showed some promising results for mapping cytokine concentrations to pathogens. Particularly, concentrations of IL-10, IFN-γ, IL-6, and TNF-α have been described over the course of experimental and natural intramammary infections and discussed as potential markers for coliform mastitis pathogens, *S. aureus* (Kim et al., 2011; Šerstņova et al., 2022), and non-*aureus* staphylococci (Kawecka-Grochocka et al., 2021). Despite promising initial results, the potential use of cytokine and chemokine concentrations in milk as biomarkers for mastitis remains underexplored. One reason for this gap in knowledge is the lack of well-described bovine reagents and sensitive, high-throughput assays to measure cytokines and chemokines in bovine milk across a broad concentration range across large sample sets.

Fluorescent bead-based multiplex assays (multiplex assays) provide a sensitive method for detection of secreted proteins with potential for multiplexing targets, facilitating high-throughput performance and timely analysis of large data sets (Christopher-Hennings et al., 2013). We recently validated a bovine cytokine multiplex assay for the detection of IL-10, IFN-γ, and TNF-α in bovine plasma, serum, and peripheral blood mononuclear cell culture supernatants (Sipka et al., 2022, 2024). The assay uses only mAb, which were extensively tested. The validation included the characterization of each mAb by flow cytometry for specific cytoplasmatic detection of the native cytokine in bovine immune cells, in addition to cross-reactivity testing for exclusion of nonspecific binding of the mAb with other sample proteins. Furthermore, the assay uses recombinant protein standards produced by mammalian cell lines to ensure high structural similarity to the respective native protein.

Our first objective was to test the suitability of milk as specimen for the bovine cytokine multiplex assay using serial dilutions of milk samples from mastitic quarters and recombinant proteins. Our second objective was to measure concentrations of IL-10, TNF-α, and IFN-γ in quarter milk samples with different bacteriological culture outcomes to investigate whether distinct cytokine patterns can be associated with common mastitis pathogens.

Sample size calculation was performed with JMP Pro (version 17, SAS Institute Inc., Cary, NC) based on IFN-γ concentrations reported for experimental *E. coli* mastitis (Sipka et al., 2013). Assuming a minimum detectable difference of 1.2 ng/mL, SD of 0.7, an α-level of 0.05, and a power of 0.95, 45 samples per group were required. To account for an anticipated attrition rate of 10%, the target sample size was increased to 250 milk samples before exclusions.

We enrolled quarter milk samples from routine submissions for mastitis diagnostics to Quality Milk Production Services at the Animal Health Diagnostic Center, Cornell University (Ithaca, NY). The quarter samples were collected and submitted by farm personnel from 17 commercial dairy farms located in central New York. Reasons for submission were clinical mastitis cases or high SCC (subclinical mastitis).

All milk samples were cultured at the bacteriology laboratory of the Animal Health Diagnostic Center, following standard procedures adapted from Dohoo et al. (2011). Cultures were considered positive in cases of ≥1 cfu of *S. aureus* or ≥3 cfu for all other organisms. Subsequently, a representative colony was submitted for MALDI-TOF analysis using the MALDI Biotyper Microflex LT (Bruker Daltonics, Billerica, MA). Adaption and maintenance of the MALDI-TOF library, sample preparation, and interpretation of the MALDI score were performed as described by Randall et al. (2015). In brief, the instrument reports a logarithmic score between 0 and 3, quantifying similarity to known database entries. A log (score) ≥1.7 was the threshold for genus-level identification, and a log (score) ≥2.0 was the set threshold for a match at the species level. If ≥3 phenotypically different organisms were present, the sample was considered contaminated and no MALDI-TOF analysis was performed.

We enrolled a total of 184 quarter samples from 179 individual cows (samples from duplicate cows were from different quarters and dates) between February and June 2025. Samples from the following pathogen groups were collected: coliform bacteria (herein termed the coliform group, comprising *E. coli* and *Klebsiella* spp.; n = 51), bacteria of the *Streptococcus* family (Strep group: *S. dysgalactiae, S. uberis, S. agalactiae, Lactococcus* spp.; n = 53), and *S. aureus* (n = 36). We also included quarter samples that did not show bacterial growth after 48-h aerobic culture (no growth; n = 44).

Additionally, we collected 55 samples from healthy quarters (healthy: California Mastitis Test negative, SCC <200,000 cells/mL on quarter level, no growth after 48-h aerobic culture) at the Cornell University Teaching Dairy (Ithaca, NY) within the same time frame as control samples. Milk sample collection in healthy cows was approved by the Institutional Animal Care and Use Committee at Cornell University (IACUC protocol 2024-0158). Somatic cell counts for healthy quarter samples were determined by Dairy One milk testing services (Dairy One, Ithaca, NY) using a Fossomatic flow cytometric instrument (Foss Analytical).

Enrolled quarter milk samples were centrifuged at 500 × *g* for 10 min at 4°C. The fat layer was removed, and an aliquot of the clot and cell-free milk whey was stored at −80°C until analysis in the bovine cytokine multiplex assay.

The bovine cytokine multiplex assay was performed as previously described (Sipka et al., 2024) with a modification for milk whey samples. Briefly, a mixture of beads coupled with mAb for IL-10, IFN-γ, and TNF-α was prepared in assay buffer (**PBN**, PBS, 1% BSA, 0.05% sodium azide) with a final concentration of 5 × 10^3^ beads each. The cytokine standard mix containing recombinant fusion proteins for all 3 targets was prepared in 5-fold dilutions in PBN. Milk whey samples were diluted 1:5 in PBN and standards, diluted samples, or PBN (blank values) were added to 96-well filter plates followed by the bead mixture. After 30-min incubation at room temperature in the dark, the plates were washed 3 times with a plate washer. These conditions were used for all following incubation and wash steps. Subsequently, the mixture of biotinylated mAb for all 3 targets was added. After another incubation and wash step, the reporter dye streptavidin R-PE was added, followed by a final incubation and washing. Plates were read in an automated reader (BioPlex 200, BioRad). Median fluorescence intensity was recorded, and the standard curves were fitted with a 5-parameter logistic regression by the BioPlex software (BioPlex Manager 6.2, BioRad). Lower limits of detection (**LOD**) for the cytokine assay were 8 pg/mL for all analytes. For samples below LOD, we assumed a concentration of ≤4 pg/mL.

Data were analyzed using the R statistical software package v4.4. 0 (https://www.R-project.org/) and packages readxl (Wickham and Bryan, 2023), ggplot (Wickham, 2016), FSA (Ogle et al., 2025), car (Fox and Weisberg, 2019), and multcomp (Hothorn et al., 2008) in R studio v2024.12.1 (Posit Software, PBC, Boston, MA). Median and interquartile range (**IQR**) were calculated and figures were created with GraphPad Prism version 10.6.1 for Windows (GraphPad Software, Boston, MA). Samples were treated as independent observations in the statistical analysis. To determine differences in cytokine concentrations among different bacteriological culture results, we used Kruskal-Wallis tests adjusted with the Holm method for multiple comparisons. Comparisons with adjusted *P*-value ≤0.05 were considered significant.

We first tested the suitability of milk as a sample matrix in the bovine cytokine assay. We performed serial dilutions of milk samples from quarters identified with coliform bacteria, as we expected high concentrations of the target analytes in these samples. The serial dilutions of the milk samples were parallel to serial dilutions of the recombinant proteins used for the standard curve, indicating that the assay can accurately quantify IL-10, TNF-α, and IFN-γ in milk samples within the linear detection range of the assay (Figure 1).

**Figure 1:**
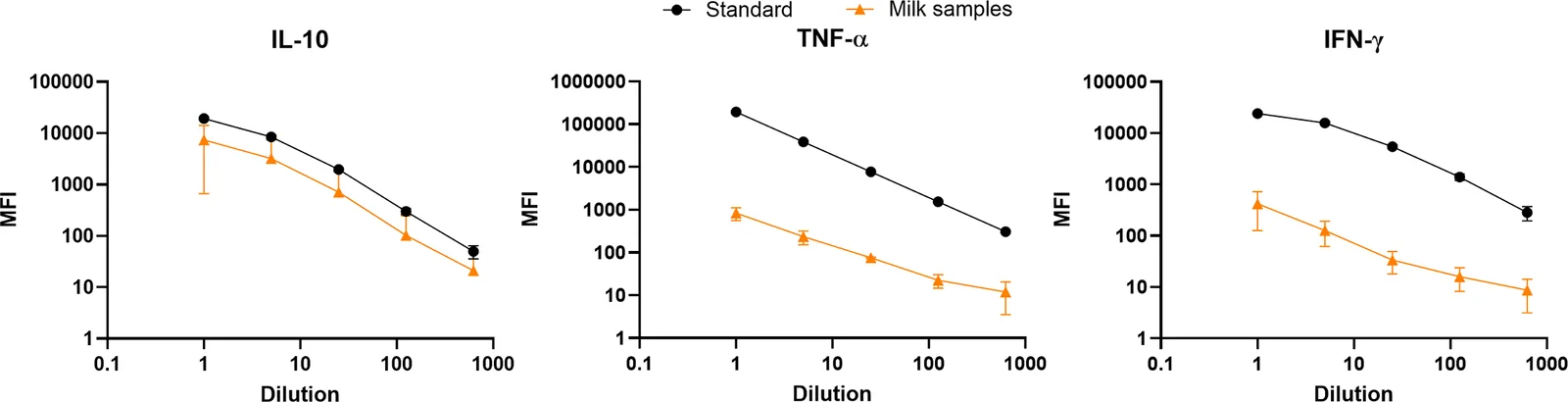
Serial dilutions of milk samples from mastitic quarters and standards within the linear quantification range of the bovine cytokine multiplex assay. Serial dilutions (1:5) were performed in assay buffer, with quarter milk samples identified with coliform bacteria (*Escherichia coli*, *Klebsiella*, n = 4) and recombinant standards for each target (IL-10, TNF-α, and IFN-γ). Graphs show median fluorescence intensity (MFI) ± SD by dilution factor for milk samples (triangles) and recombinant protein standards (circles) for IL-10, TNF-α, and IFN-γ.

Quarter milk from quarters enrolled as healthy had a median SCC of 16,000 cells/mL (IQR: 9,000–23,000 cell/mL) with a maximum of 67,000 cells/mL, which was substantially below the enrollment threshold of 200,000 cells/mL.

We detected cytokine concentrations above the LOD of the multiplex assay in healthy samples. Out of 55 healthy samples 35 (64%) were within range of the assay for IL-10, 25 (45%) for TNF-α, and 16 (29%) for IFN-γ. Of the quarter samples enrolled with no growth (n = 44), 32 (73%) had detectable concentrations of IL-10, 16 (36%) for TNF-α, and 18 (41%) for IFN-γ. Quarter samples that were identified with the coliform or Strep groups had the highest proportions of in-range results. For quarters identified with the coliform group (n = 51), proportions of in-range results were highest, with 100% (51) for IL-10, 98% (50) for TNF-α, and 82% (42) for IFN-γ. Samples identified with the Strep group (n = 53) had 92% (49) of results in range for IL-10, 75% (40) for TNF-α, and 92% (49) for IFN-γ. Among samples enrolled with *S. aureus* (n = 36), 81% (29) had in-range results for IL-10, 53% (19) for TNF-α, and 39% (14) for IFN-γ. Distribution of cytokine concentrations can be found in Figure 2A.

**Figure 2:**
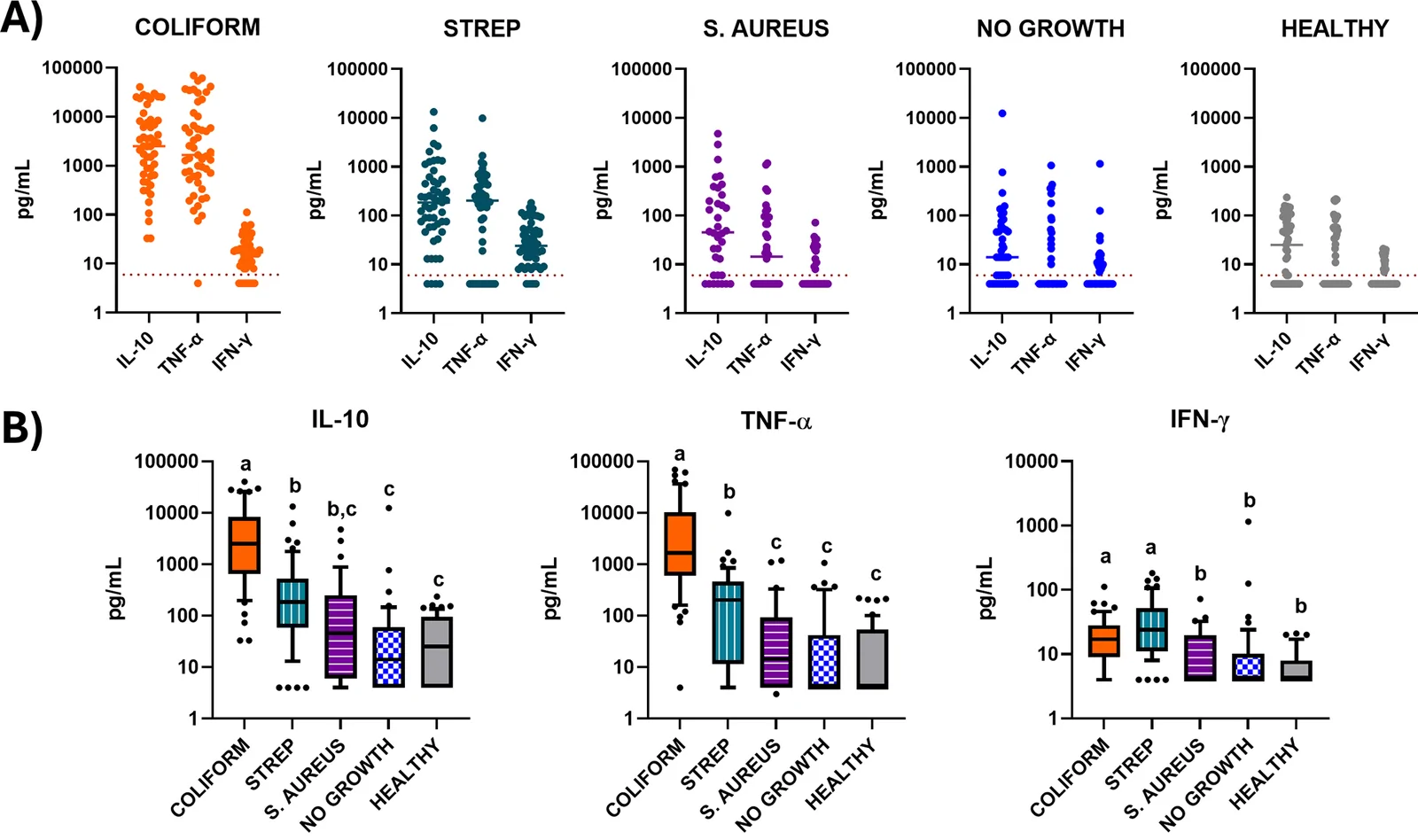
Cytokine profiles of enrolled quarter samples with different bacteriological culture outcomes. Concentrations (pg/mL) of IL-10, TNF-α, and IFN-γ were measured with a fluorescent bead-based multiplex assay in quarter milk samples with different bacteriological culture outcomes: coliform bacteria (coliform group, comprising *Escherichia coli* and *Klebsiella* spp.; n = 51), bacteria from the *Streptococcus* family (Strep: *Streptococcus uberis, Streptococcus dysgalactiae*, and *Lactococcus* spp.; n = 53), *Staphylococcus aureus* (*S. aureus*; n = 36), samples from quarters with mastitis an no growth after 48-h aerobic culture (no growth; n = 44), and quarter milk samples from healthy quarters (healthy: California Mastitis Test negative, SCC <200,000 cells/mL, no growth after 48-h aerobic culture; n = 55). (A) Cytokine concentrations, shown by bacteriological culture outcome as dot plots with median. (B) Cytokine concentrations for different bacteriological outcomes, compared by cytokine. Data are shown as box and whisker plots. The box extends from the 25th to the 75th percentile; the median is shown as a horizontal midline. The whiskers are drawn down to the 10th percentile and up to the 90th percentile. Values below and above the whiskers are drawn as individual points. Significant differences between concentrations are indicated by different lowercase letters (a–c; *P* ≤ 0.05).

Median and IQR for all culture outcomes and analytes are summarized in Table 1. When comparing cytokine concentrations by culture outcome (Figure 2B), we found that the coliform group had the highest IL-10 and TNF-α concentrations compared with all other culture outcomes (*P* < 0.001 for both cytokines and all comparisons). Intramammary infections with coliform bacteria typically cause clinical mastitis (Fredebeul-Krein et al., 2022). Our study showed expected cytokine patterns for the coliform samples. The cytokines TNF-α and IL-10 are secreted in milk within hours after infections with coliform bacteria (Herry et al., 2017). In experimental mastitis challenges with *E. coli*, TNF-α peaked at 16 h and IL-10 at 24 h after infection (Sipka et al., 2013; Herry et al., 2017). Clinical signs in coliform mastitis appear ∼12 to 16 h after infection (Sipka et al., 2013). Cows are milked every 8 to 12 h. It is therefore likely that the samples enrolled as coliform in this study were collected in the early onset of TNF-α and IL-10 secretion. Concentrations of IFN-γ were higher in the coliform group compared with the *S. aureus* (*P* = 0.002), no growth (*P* < 0.001), and healthy groups (*P* < 0.001) but were overall much lower than IL-10 and TNF-α in coliform samples. In experimental infections with *E. coli*, IFN-γ was measured in peak concentrations later than IL-10 and TNF-α (Sipka et al., 2013). It is possible that the samples we enrolled for the coliform group were collected before INF-γ concentrations reached peak levels. However, Hisaeda et al. (2001) also observed lower IFN-γ than TNF-α concentrations in whey of cows with natural *E. coli* mastitis.

**Table 1:** Median cytokine concentrations plus interquartile ranges (IQR) for IL-10, TNF-α, and IFN-γ in quarter milk samples with different bacteriological culture outcomes

Culture group	Median concentration, pg/mL (IQR)		
	IL-10	TNF-α	IFN-γ
Coliform: *Escherichia coli, Klebsiella* spp.; n = 51	2,501	1,658	17
	(645–8,338)	(604–10,128)	(9–28)
Strep: *Streptococcus uberis, Streptococcus dysgalactiae, Lactococcus* spp.; n = 53	186	202	24
	(59–525)	(12–458)	(11–52)
*S. aureus*: *Staphylococcus aureus*; n = 36	46	15	≤4
	(≤4–249)	(≤4–92)	(≤4–20)
No growth: Mastitic quarter,^1^ no bacterial growth; n = 44	14	≤4	≤4
	(≤4–59)	(≤4–42)	(≤4–10)
Healthy: Healthy quarter,^2^ no bacterial growth; n = 55	25	≤4	≤4
	(≤4–95)	(≤4–54)	(≤4–8)

1 Quarter sample submitted for pathogen detection to Animal Health Diagnostic Center, Cornell University, Ithaca, NY.

2 Normal milk, SCC <200,000 cells/mL.

Samples identified with the Strep group had higher TNF-α and IFN-γ concentrations compared with *S. aureus* (TNF-α *P* = 0.005, IFN-γ *P* < 0.001), no growth (*P* < 0.001 for both analytes), and healthy samples (*P* < 0.001 for both analytes), and higher IL-10 concentrations compared with no growth (*P* < 0.001) and healthy quarters (*P* < 0.001). We included a wider range of organisms in this outcome (*S. uberis, S. dysgalactiae*, and *Lactococcus* spp.). These pathogens are known to cause mild to severe clinical or subclinical mastitis and are often combined when making mastitis management decisions, due to their microbiological similarities and responsiveness to treatment (Werner et al., 2014; Cameron et al., 2016; Fredebeul-Krein et al., 2022). In experimental infections with *S. uberis*, similar concentrations for TNF-α were found between 66 and 168 h after infection, which was also the period with the highest SCC and cfu concentrations in milk (Rambeaud et al., 2003). Others, however, have described substantial variation between *S. uberis* strains, and information on cytokine concentrations induced by *S. dysgalactiae* and *Lactococcus* spp. is scarce (Srithanasuwan et al., 2024).

Quarters identified with *S. aureus* did not differ in cytokine concentrations from no growth or healthy quarters. The cytokine with the highest concentration for this outcome was IL-10. Intramammary infections caused by *S. aureus* are often subclinical, and low to nondetectable concentrations of the proinflammatory cytokines TNF-α and IFN-γ appear plausible. We did not collect clinical severity of the cases for this study. For future studies it could be interesting to investigate whether higher concentrations of proinflammatory cytokines are associated with higher clinical severity or cure.

Samples with no growth were not significantly different from healthy samples. The reason for the lack of bacterial growth after 48 h of aerobic culture is difficult to explain. Possibilities include that bacteria are below the detectable threshold of the culture method, potentially due to elimination of the invading pathogen by the host's immune system, or that the causative bacteria do not grow under aerobic conditions. Our results showed cytokine concentrations similar to milk from healthy quarters, which indicates no strong stimulation of any of the 3 cytokines in these quarters at the time of sample collection.

Our choice to use a convenience sample of routinely submitted field samples brought some limitations for this study. Information on severity of clinical signs, previous treatment, or resolution of infection was not available to us. This most likely increased variation of cytokine concentrations for each culture outcome. Further studies should include this information, to facilitate association of cytokine concentrations with clinical severity, treatment, and outcome of infection. For this study we decided on broad pathogen categories that correspond with current mastitis management strategies. Future studies should also consider a more narrowly defined pathogen category, to investigate differences between bacterial species or strains. It should also be noted that the multiplex assay requires specific and costly laboratory equipment as well as a controlled laboratory environment and is not suited for on-farm evaluation of samples. The assay should be considered as a tool for finding a suitable biomarker for mastitis, due to its high sensitivity and potential for high throughput of samples for screening.

In this study, we validated the use of milk in the bovine multiplex assay. Using this assay, we successfully screened quarter milk samples with different bacteriological culture outcomes and found differences between some of the outcomes. We also observed high variation between individual samples for all outcomes and all cytokines. Future studies should include more detailed information on mastitis cases and focus on individual mastitis pathogens on species or strain levels. The bovine multiplex assay may be a useful tool for the quantification of IL-10, TNF-α, and IFN-γ in quarter milk samples, facilitating high-throughput screening for identifying potential biomarkers for bovine mastitis.
